# Supplementary material for: Vitamin D Deficiency Is Significantly Associated with Retinopathy in Type 2 Diabetes Mellitus: A Case-Control Study
Source: Nutrients. 2021 Dec 25;14(1):84. doi: 10.3390/nu14010084 (PMC8746564; doi:10.3390/nu14010084)
Supplement: Supplementary file 1 [file nutrients-14-00084-s001.zip › nutrients-1494397-supplementary.pdf]

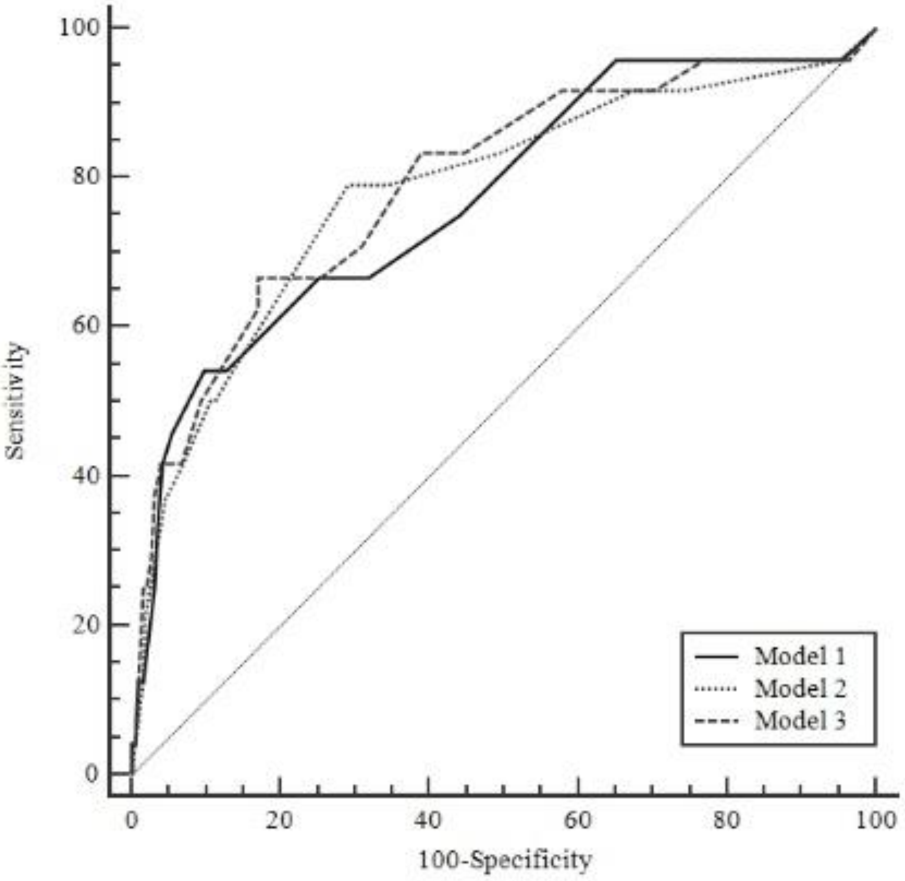

Figure S1. Area under de curve of the 3 models. Pairwise comparison of ROC curves: DeLong test.

Model 1 vs Model 2: p= 0.779; Model 1 vs Model 3: p= 0.366 Model 2 vs Model 3: p= 0.455

Table S1. Initial model, Model 0, and steps in conditional backward selection variables.

		B	Standar error	Wald	df	pvalue.	OR	95% CI Inferior	Superior
**Model 0**	Age	.028	.031	.836	1	.381	1.029	.968	1.093
	BMI <25 (reference)			2.400	2	.301			
	Overweight 25 - 29.99	-.168	.787	.046	1	.830	.845	.181	3.947
	Obesity (> 30)	-.960	.847	1.285	1	.257	.383	.073	2.014
	Years since diagnosis: < 5 years (reference)			6.307	5	.277			
	6-9 years	1.834	1.166	2.471	1	.116	6.257	.636	61.549
	10-14 years	1.872	1.122	2.785	1	.095	6.502	.721	58.612
	15-19 years	0.741	1.275	.338	1	.561	2.098	.172	25.517
	20-24 years	.226	1.971	.013	1	.909	1.253	.026	59.606
	≥ 25 years	2.386	1.313	3.302	1	.069	10.865	.829	142.406
	Treatment of diabetes: diet (reference)			7.542	3	.056			
	OAD	.999	1.134	.776	1	.378	2.716	.294	25.073
	Insulin	3.066	1.418	4.674	1	.031	21.461	1.332	345.823
	OAD + Insulin	2.317	1.276	3.299	1	.069	10.147	.832	123.690
	HBA1C: < 7% (REFERENCE)			.620	3	.892			
	7-8%	.068	.649	.011	1	.916	1.070	.300	3.819
	8,01-10%	-.343	.949	.131	1	.718	.710	.110	4.557
	> 10%	1.007	2.191	.211	1	.646	2.738	.037	200.745
	HBP: normotense (reference)			3.255	2	.196			
	STAGE 1	1.038	.619	2.808	1	.094	2.823	.839	9.503
	STAGE 2	1.217	.922	1.742	1	-.187	3.377	.554	20.578
	Cardiovascular events (number)	-.146	.360	.164	1	.685	.864	.426	1.751
	Renal faliure	.510	.563	.820	1	.365	1.666	.552	5.025
	25(OH) D (<16 ng/ml)	.783	.522	2.247	1	.134	2.189	.786	6.094
	1,25 (OH)2 D (< 29 pg/ml)	.801	.541	2.193	1	.139	2.228	.772	6.432
	Constant	-8.697	2.772	9.843	1	.002	.000		
Step 2	AGE	.028	.029	.943	1	.332	1.029	.972	1.089
	BMI < 25 (reference)			2.972	2	.226			
	Overweight 25 - 29.99	-.310	.737	.177	1	.674	.734	.173	3.110
	Obesity (> 30)	-1.119	.782	2.045	1	.153	.327	.071	1.514
	Years_evolución diabetes (≤ 5 years)			6.064	5	.300			
	6-9 years	1.842	1.166	2.493	1	.114	6.307	.641	62.022
	10-14 years	1.861	1.118	2.772	1	.096	6.430	.719	57.508
	15-19 years	.753	1.271	.351	1	.554	2.123	.176	25.632
	20-24 years	.441	1.689	.068	1	.794	1.554	.057	42.529
	≥ 25 years	2.270	1.257	3.260	1	.071	9.682	.824	113.810
	Treatment of diabetes: diet (reference)			10.637	3	.014			
	OAD	1.035	1.124	.848	1	.357	2.815	.311	25.479

	Insulin	3.141	1.304	5.800	1	.016	23.120	1.794	297.894
	OAD + Insulin	2.251	1.194	3.551	1	.060	9.496	.914	98.691
	HBP: normotense (reference)			3.283	2	.194			
	STAGE 1	.981	.603	2.644	1	.104	2.668	.818	8.704
	STAGE 2	1.291	.897	2.074	1	.150	3.637	.627	21.081
	Cardiovascular events (number)	-.173	.346	.251	1	.616	.841	.427	1.657
	Renal faliure	.533	.550	.939	1	.333	1.703	.580	5.002
	Niveles 25 (OH) D (≤ 16 ng/ml)	.802	.519	2.384	1	.123	2.229	.806	6.167
	1,25 (OH)2 D (< 29 pg/ml)	.748	.533	1.971	1	.160	2.113	.744	6.006
	Constant	-8.527	2.652	10.338	1	.001	.000		
Step 3	AGE	.025	.028	.774	1	.379	1.025	.970	1.084
	BMI < 25 (reference)			2.921	2	.232			
	Overweight 25 - 29.99	-.267	.730	.134	1	.714	.765	.183	3.199
	Obesity (> 30)	-1.081	.775	1.948	1	.163	.339	.074	1.548
	Years since diagnosis: < 5 years (reference)			6.214	5	.286			
	6-9 years	1.812	1.161	2.434	1	.119	6.122	.629	59.636
	10-14 years	1.877	1.114	2.840	1	.092	6.535	.736	57.984
	15-19 years	.740	1.265	.342	1	.558	2.097	.176	25.042
	20-24 years	.466	1.662	.079	1	.779	1.594	.061	41.374
	≥25 years	2.289	1.252	3.341	1	.068	9.861	.848	114.720
	Treatment of diabetes: diet (reference)			10.405	3	.015			
	OAD	1.056	1.128	.875	1	.349	2.874	.315	26.245
	Insulin	3.098	1.305	5.636	1	.018	22.160	1.717	286.065
	OAD + Insulin	2.238	1.199	3.485	1	.062	9.377	.848	114.720
	HBP: normotense (reference)			3.365	2	.186			
	STAGE 1	.987	.607	2.645	1	.104	2.682	.817	8.810
	STAGE 2	1.334	.894	2.230	1	.135	3.797	.659	21.878
	Renal faliure	.496	.544	.831	1	.362	1.643	.565	4.775
	25(OH) D (< 16 ng/ml)	.811	.518	2.449	1	.118	2.250	.815	6.210
	1,25 (OH)2 D (< 29 pg/ml)	.742	.532	1.946	1	.163	2.101	.740	5.963
	Constant	-8.420	2.639	10.180	1	.001	.000		
Step 4	BMI < 25 (reference)			2.952	2	.229			
	Overweight 25 - 29.99	-.293	.734	.159	1	.690	.746	.177	3.144
	Obesity (> 30)	-.1.098	.777	1.998	1	.157	.334	.073	1.529
	Years since diagnosis: < 5 years (reference)			6.362	5	.273			
	6-9 years	1.767	1.147	2.372	1	.124	5.852	.618	55.439
	10-14 years	1.866	1.105	2.854	1	.091	6.465	.742	56.354
	15-19 years	.817	1.253	.426	1	.514	2.265	.194	26.374
	20-24 years	.357	1.687	.045	1	.832	1.429	.052	38.985
	≥ 25 years	2.371	1.245	3.624	1	.057	10.704	.932	122.895
	Treatment of diabetes: diet (reference)			10.563	3	.014			
	OAD	1.026	1.130	.825	1	.364	2.791	.305	25.548

	Insulin	3.054	1.297	5.544	1	.019	21.209	1.669	269.579
	OAD + Insulin	2.231	1.199	3.462	1	.063	10.704	.888	97.732
	HBP: normotense (reference)			3.471	2	.176			
	STAGE 1	1.040	.603	2.975	1	.085	2.829	.868	9.225
	STAGE 2	1.282	.909	1.991	1	.158	3.605	.607	21.397
	Renal faliure	.598	.532	1.261	1	.261	1.818	.641	5.156
	25 (OH) D (≤ 16 ng/ml)	.853	.514	2.752	1	.097	2.346	.857	6.423
	1,25 (OH)2 D (< 29 pg/ml)	.772	.532	2.105	1	.147	2.164	.763	6.142
	Constant	-6.676	1.668	16.023	1	.000	.001		
Step 5	BMI < 25 (reference)			3.039	2	.219			
	Overweight 25 - 29.99	-.302	.728	.172	1	.679	.739	.177	3.083
	Obesity (> 30)	-1.112	.772	2.078	1	.149	.329	.072	1.492
	Years since diagnosis: < 5 years (reference)			6.386	5	.270			
	6-9 years	1.821	1.149	2.512	1	.113	6.176	.650	58.678
	10-14 years	1.899	1.106	2.950	1	.086	6.680	.765	58.342
	15-19 years	1.004	1.238	.658	1	.417	2.730	.241	30.893
	20-24 years	.619	1.633	.144	1	.704	1.858	.076	45.557
	≥ 25 years	2.529	1.227	4.251	1	.039	12.546	1.133	138.906
	Treatment of diabetes: diet (reference)			11.052	3	.011			
	OAD	1.051	1.122	.877	1	.349	2.860	.317	25.791
	Insulin	3.046	1.284	5.627	1	.018	21.031	1.698	260.524
	OAD + Insulin	2.311	1.186	3.799	1	.051	10.089	.987	103.119
	HBP: normotense (reference)			5.082	2	.079			
	STAGE 1	1.212	.578	4.390	1	.036	3.359	1.081	10.437
	STAGE 2	1.461	.885	2.721	1	.099	4.309	.760	24.438
	25 (OH) D (≤ 16 ng/ml)	.901	.506	3.174	1	.075	2.462	.914	6.633
	1,25 (OH)2 D (< 29 pg/ml)	.781	.528	2.192	1	.139	2.184	.776	6.143
	Constant	-6.720	1.661	16.373	1	.000	.001		
Step 6	Years since diagnosis: < 5 years (reference)			6.282	5	.280			
	6-9 years	1.672	1.133	2.177	1	.140	5.322	.578	49.034
	10-14 years	1.782	1.099	2.627	1	.105	5.940	.689	51.237
	15-19 years	1.099	1.225	.805	1	.369	3.002	.272	33118
	20-24 years	.656	1.563	.0176	1	.675	1.926	.090	41.260
	≥ 25 years	2.562	1.214	4.451	1	.035	12.956	1.199	139.949
	Treatment of diabetes: diet (reference)			9.873	3	.020			
	OAD	.784	1.093	.514	1	.473	2.189	.257	18.639
	Insulin	2.622	1.237	4.492	1	.034	13.759	1.218	155.419
	OAD + Insulin	2.036	1.147	3.151	1	.076	7.658	.809	72.500
	HBP: normotense (reference)			4.930	2	.085			
	STAGE 1	1.157	.567	4.157	1	.041	3.179	1.046	9.665
	STAGE 2	1.477	.879	2.824	1	.093	4.381	.782	24.535
	25 (OH) D (≤ 16 ng/ml)	.862	.491	3.091	1	.079	2.369	.906	6.196

	1,25 (OH)2 D (< 29 pg/ml)	.845	.518	2.658	1	.103	2.328	.843	6.432
	Constant	-6.940	1.599	18.847	1	.000	.001		
Step 7	Treatment of diabetes: diet (reference)			15.612	3	.001			
	OAD	.636	1.075	.350	1	.554	1.889	.230	15.528
	Insulin	2.678	1.183	5.127	1	.024	14.553	1.433	147.776
	OAD + Insulin	2.231	1.108	4.054	1	.044	9.305	1.061	81.605
	HBP: normotense (reference)			3.863	2	.145			
	STAGE 1	.978	.526	3.459	1	.063	2.658	.949	7.448
	STAGE 2	1.156	.877	1.740	1	.187	3.177	.570	17.708
	25 (OH) D (≤ 16 ng/ml)	.825	.476	2.997	1	.083	2.281	.897	5.801
	1,25 (OH)2 D (< 29 pg/ml)	.768	.505	2.315	1	.128	2.155	.801	5.797
	Constant	-5.203	1.154	20.320	1	.000	.006		
				4.945	2	.084			
		1.025	.529	3.765	1	.052	2.788	.990	7.857
Step 8	Treatment of diabetes: diet (reference)			17.419	3	.001			
	OAD	.669	1.069	.391	1	.532	1.952	.240	15.858
	Insulin	2.834	1.167	5.900	1	.015	17.011	1.728	167.429
	OAD + Insulin	2.285	1.101	4.310	1	.038	9.823	1.136	84.916
	HBP: normotense (reference)			4.642	2	.098			
	STAGE 1	1.044	.528	3.916	1	.048	2.840	1.010	7.988
	STAGE 2	1.340	.849	2.490	1	.115	3.819	.723	20.179
	25 (OH) D (≤ 16 ng/ml)	1.029	.458	5.054	1	.025	2.799	1.141	6.866
	Constant	-4.968	1.124	19.523	1	.000	.007		

Table S2. Effect of treatment with insulin controlled for the variables age, years since onset, vitamin D, glycemic control, and control of HBP.

Variable	P value	OR	IC 95%
Age	0.249	1.03	0.98 – 1.09
Years since diagnosis	0.679	1.02	0.95 – 1.09
Treatment DM diet/OAD (reference)	0.044		
Insulin	0.033	5.76	1.16 – 28.69
OAD + Insulin	0.029	4.21	1.16 – 15.29
25(OH) D < 16 ng/ml	0.162	1.97	0.76 – 5.12
1,25$(OH)_2$ D < 29 pg/ml	0.103	2.33	0.84 – 6.47
Glycemic control			
HbA1c < 7 (reference)	0.829		
HbA1c 7-7.9	0.607	1.37	0.41 – 4.57
HbA1c 8-10	0.940	1.07	0.20 – 5.79
HbA1c >10	0.418	4.56	0.12 – 179.05
HBP normal (reference)	0.222		
Stage 1	0.104	2.44	0.83 – 7.15
Stage 2	0.263	2.75	0.47 – 16.17
Constant	0.000	0.01	

Table S3. Risk of DR (crude OR) according to cut-off for 25(OH)D.

	**p value**	**OR**	**95% CI**
Categories 25(OH)D and 1,25(OH)$_2$D composite			
25(OH)D >16 ng/ml & 1,25(OH)$_2$D >29 pg/ml	0.011		
25(OH)D >16 * & 1,25(OH)2D ≤29 **	0.882	1.135	0.21 – 6.04
25(OH)D ≤16 * & 1,25(OH)2D >29 **	0.341	1.853	0.52 – 6.61
25(OH)D ≤16 * & 1,25(OH)2D ≤29 **	0.003	5.212	1.76 – 15.42
SEIOMM cutoff threshold			
25(OH)D >30 ng/ml	0,032		
25(OH)D <10 ng/ml	0.042	3.757	1.58 – 13.46
25(OH)D 10-30 ng/ml	0.984	0.989	0.36 – 2.76
IOM cutoff threshold			
25(OH)D <20 ng/ml	0.046	2.285	1.01 – 5.16
Associated value of Youden index*			
25(OH)D <16 ng/ml	0.021	2.427	1.14 – 5.16

SEIOMM: Sociedad Española de Investigación Ósea y del Metabolismo MineralL. IOM: Institute of Medicine. *Associated value of Youden index in present study.
